# Transdifferentiation of cervical squamous cell carcinoma with ERBB2 amplification to adenocarcinoma: whole genome sequence analysis and successful control by anti-HER2 therapy

**DOI:** 10.1038/s44276-023-00015-9

**Published:** 2023-09-04

**Authors:** Hiroaki Ikushima, Kiyoshi Yamaguchi, Yoichi Furukawa, Seiya Imoto, Hirotomo Koda, Takuro Mizukami, Teppei Morikawa, Keita Uchino

**Affiliations:** 1 Department of Medical Oncology, NTT Medical Center Tokyo, Tokyo, Japan; 2 Division of Clinical Genome Research, Advanced Clinical Research Center, The Institute of Medical Science, The University of Tokyo, Tokyo, Japan; 3 Division of Health Medical Intelligence, Human Genome Center, The Institute of Medical Science, The University of Tokyo, Tokyo, Japan; 4 Department of Diagnostic Pathology, NTT Medical Center Tokyo, Tokyo, Japan

## Abstract

Cancer cells sometimes transdifferentiate into different histological type(s) and tumors with multiple histological types can share a common ancestor cell. However, diagnosis of the origin of multiple tumor lesions with different histological features remains a clinical challenge. A 45-year-old woman with a history of cervical squamous cell carcinoma (CeSq) presented with abdominal pain and vomiting. A surgical operation revealed an ileal tumor and a peritoneal nodule with a small amount of ascites. A histological examination of the ileal tumor demonstrated squamous cell carcinoma, which was consistent with metastasis of cervical cancer, while that of the nodule and ascites showed adenocarcinoma. Whole genome sequencing (WGS) of the CeSq, ileal squamous cell carcinoma (SiSq), and peritoneal adenocarcinoma (PeAd) demonstrated that *ERBB2* was commonly amplified in all lesions. Additionally, HPV-16 genome sequences were identified at identical genomic loci in these lesions. A trajectory analysis corroborated that SiSq and PeAd had a shared origin and developed simultaneously at each metastatic site. These results indicate that a subpopulation of the CeSq had transdifferentiated into adenocarcinoma in our patient. Anti-HER2 therapy showed marked effects on the recurrent disease. Our case demonstrates the plasticity of tumor cells and reinforces the potential roles of WGS in the implementation of precision oncology.

## Background

In the clinical setting, when multiple tumor lesions are detected on imaging, histopathological findings may give us some clues as to whether they are independent simultaneous tumors or whether one lesion metastasized to other lesions. However, recent studies have revealed that cancer cells have the ability to transdifferentiate into different histological type(s), and that tumors with different histological types may have a common ancestor cell [1–3]. These findings make it more difficult to accurately diagnose the origin of multiple tumor lesions found in unrelated organs.

Amplification of the *ERBB2* gene, which leads to the overproduction of HER2 protein, is associated with the development and progression of certain types of cancer, particularly breast and gastric cancer [4–6]. The introduction of targeted therapies that specifically target the HER2 pathway, such as trastuzumab, has greatly improved outcomes for patients with HER2-positive cancers [7–9].

We herein report the case of a 45-year-old woman with cervical squamous cell carcinoma and peritoneal dissemination of adenocarcinoma. A whole genome sequencing (WGS) analysis revealed that the squamous cell carcinoma lesion and the adenocarcinoma lesion shared genetic patterns such as *ERBB2*-amplification and integration of the human papilloma virus 16 (HPV-16) genome and that they originated from common ancestor cells. Her metastatic recurrent disease was successfully controlled by anti-HER2 therapy. The fact that adenocarcinoma can share its origin with squamous cell carcinoma is clinical evidence of tumor cell plasticity.

## Methods

### Study design

Appropriate written informed consent was obtained for publication of this case report and accompanying images. The study protocol was reviewed and approved by the ethics committee of NTT Medical Center Tokyo (approval number: 21-120) and the Institute of Medical Science, the University of Tokyo (2020-78).

### Whole genome sequencing

Genomic DNA was isolated from control saliva and formalin-fixed paraffin-embedded (FFPE) tumor tissues using an Oragene DNA kit (OG-500, DNA Genotek, Ontario, Canada) and a GeneRead DNA FFPE Kit (Qiagen, Valencia, CA), respectively. Sequence libraries were prepared using 50 ng of the fragmented DNA and the MGIEasy Universal DNA Library Prep Kit, according to the manufacturer’s instructions (MGI Tech, Shenzhen, China). Sequencing was performed with 150 bp paired-end reads on the DNBSEQ-G400RS platform (MGI Tech). Fastq files were aligned to human reference sequence (hg19) using Burrows-Wheeler Aligner in NVIDIA Clara Parabricks (ver. 3.7.0.4) and bam files were created for data processing. The average sequence-read depth of CeSq, SiSq, PeAd, and saliva was 74.5×, 60.1×, 65.1×, and 58.2×, respectively. Genomon (ver. 2.6.2), an in-house pipeline constructed at the Human Genome Center, The University of Tokyo, was used for the detection of single nucleotide variants and short insertions/deletions (https://github.com/Genomon-Project). The EBcall (Empirical Bayesian mutation calling) algorithm was used to identify somatic mutations [10]. This algorithm discriminates somatic mutations from sequencing errors based on an empirical Bayesian method. In addition, CNVkit (ver 0.9.4) was applied for the detection of copy number variations from WGS data [11]. To estimate tumor cellularity, we used Sequenza (ver. 3.0.0). The evolutionary history of tumors based on somatic alterations was analyzed using the getPhyloTree function in R package MesKit (ver. 1.4.0) [12]. The phylogenetic analysis utilized neighbor-joining (NJ) algorithm, and the minimum variant allele frequency was set to 0.04. HPV fusion sites on human genome were detected using SearcHPV (ver. 1.0.10) [13].

## Case presentation

**Fig. 1:**
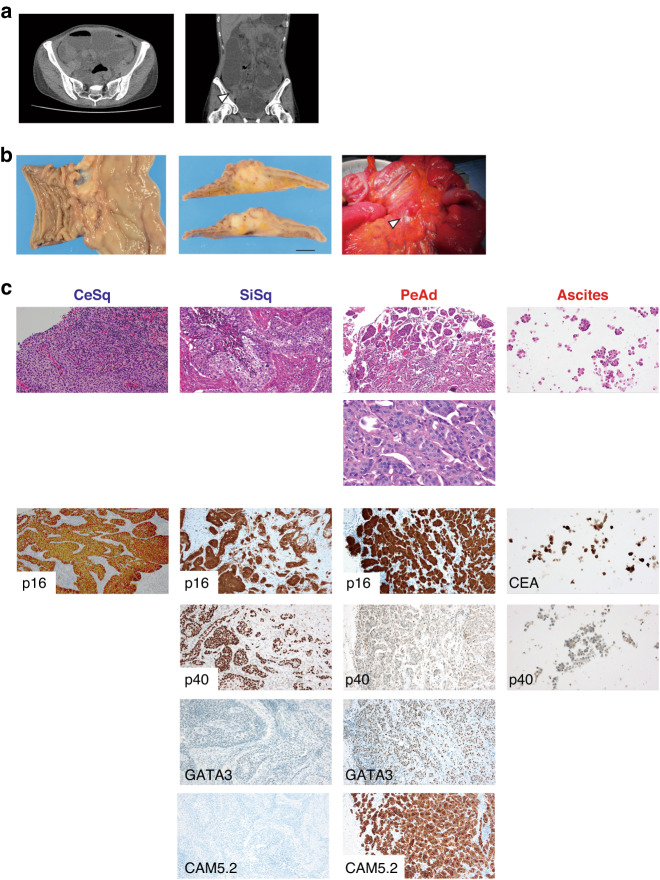
Radiological and pathological findings at the diagnosis of squamous cell carcinoma and adenocarcinoma. **a** A CT scan on admission showed stenosis of the ileum (arrowhead) and dilatation of the proximal side of the small intestine. **b** Resection of the constructed ileum (left) with a substantial white lesion (middle. Bar: 1 cm). Small peritoneal nodule (arrowhead) on the mesentery (right). **c** Hematoxylin and eosin staining (first row: low magnification, second row: high magnification) and immunohistochemistry of the collected samples. CeSq uterine cervical cancer (squamous cell carcinoma), SiSq small intestinal lesion (squamous cell carcinoma), PeAd peritoneal nodule (adenocarcinoma).

A 45-year-old woman who had undergone total hysterectomy and bilateral salpingectomy for uterine cervical cancer (squamous cell carcinoma in situ) in another hospital one year before visited our hospital due to abdominal pain and vomiting. No obvious tumor was found in the gynecological examination. Laboratory tests showed elevated levels of carcinoembryonic antigen (CEA; 12.9 ng/ml), cancer antigen 125 (CA125; 81 U/ml), and squamous cell carcinoma antigen (SCC; 2.7 ng/ml). Computed tomography (CT) revealed stenosis of the ileum and dilatation of the proximal side of the stenosis (Fig. 1a). During surgery, a substantial white lesion of approximately 30 mm in size, was found in the ileum, 80 cm from the ileocecal junction, and was exposed to the serosal surface, along with constriction of the intestine at this site (Fig. 1b). Additionally, a 5-mm peritoneal nodule on the mesentery and yellowish ascites in the abdominal cavity were observed (Fig. 1b). The ileal lesion with the constricted intestine and the peritoneal nodule were resected and histologically examined, along with the ascites.

The ileal specimen contained keratinizing squamous cell carcinoma and did not contain adenocarcinoma components (Fig. 1c). An immunohistochemical analysis revealed that tumor cells in the intestinal lesion were positive for p40 and p16, both of which were also positive in the cervical cancer cells resected one year before the operation (Fig. 1c), indicating that the ileal lesion was p16-positive squamous cell carcinoma that had metastasized from the primary cervical cancer. In contrast, the peritoneal nodule was composed of atypical epithelial cells exhibiting a micropapillary-like structure (Fig. 1c), which differed histologically from the ileal lesion or the primary cervical cancer. A cytological examination of the ascites revealed the presence of adenocarcinoma cells (Fig. 1c). An immunohistochemical analysis showed that the tumor cells in the peritoneal nodule were diffusely positive for GATA3 and CAM5.2, but negative for p40 (Fig. 1c). Although we could not exclude the possibility of the presence of an adenosquamous carcinoma, none of the uterine cervical specimen, the small intestinal specimen, or the peritoneal nodule specimen displayed a mixed histopathological phenotype of squamous cell carcinoma and adenocarcinoma. The small intestinal region was p40-positive GATA3-negative CAM5.2-negative squamous cell carcinoma while peritoneal nodule was GATA3-positive CAM5.2-positive p40-negative adenocarcinoma (Fig. 1c), suggesting the presence of two independent cancers occurring simultaneously at different organs. As the ascites was cytologically positive for adenocarcinoma, additional treatment for the adenocarcinoma was necessary. Thus, the patient underwent a whole-body survey, including positron emission tomography (PET)-CT, gastrointestinal endoscopy, and mammography, but no additional tumors were discovered. Therefore, we decided to initiate treatment for primary peritoneal adenocarcinoma [14–16], and she received 7 cycles of paclitaxel (175 mg/m^2^) + carboplatin (AUC 6) + bevacizumab (15 mg/kg) as the first-line therapy (TC + bevacizumab). After 7 cycles of TC + bevacizumab, no recurrence of ascites or other lesions were observed, and we proceeded to bevacizumab maintenance therapy.

**Fig. 2:**
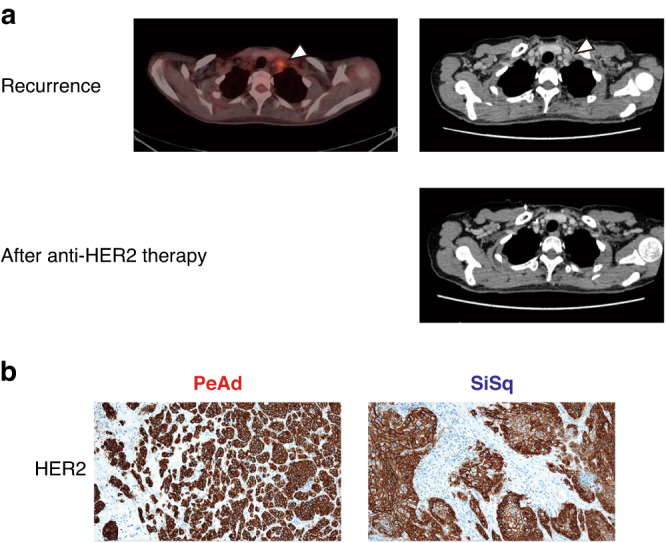
HER2 overexpression both in the adenocarcinoma lesion and the squamous cell carcinoma lesion. **a** CT and PET-CT scans revealed enlargement of the left supraclavicular lymph node (arrowhead). CT revealed that the size of the left supraclavicular lymph node decreased after three cycles of trastuzumab deruxtecan (T-DXd). **b** HER2 (clone 4B5) was highly expressed in the peritoneal nodule (PeAd) and the small intestinal lesion (SiSq).

One year after the initiation of TC + bevacizumab therapy, CT and PET-CT showed enlargement of the left supraclavicular lymph node (Fig. 2a). Elevated levels of CEA and CA125 (75.4 ng/ml and 78 U/ml, respectively) were observed, although her SCC value remained within the normal limits. In order to explore additional therapeutic options for her adenocarcinoma of unknown primary, we performed genomic profiling of the peritoneal adenocarcinoma lesion. A FoundationOne® next generation sequencing assay revealed high amplification of the *ERBB2* gene. An immunohistochemical analysis confirmed the high expression of HER2 in the peritoneal lesion (Fig. 2b). Based on these results, following two regimens of anti-HER2 antibody agents, a HER2-targeting antibody-drug conjugate, trastuzumab deruxtecan (T-DXd), was administered. After three cycles of T-DXd, CT showed shrinkage of the left supraclavicular lymph node (Fig. 2a), and the levels of tumor markers were reduced (CEA: 430.0 ng/ml to 7.3 ng/ml, CA125 409 U/ml to 11 U/ml).

**Fig. 3:**
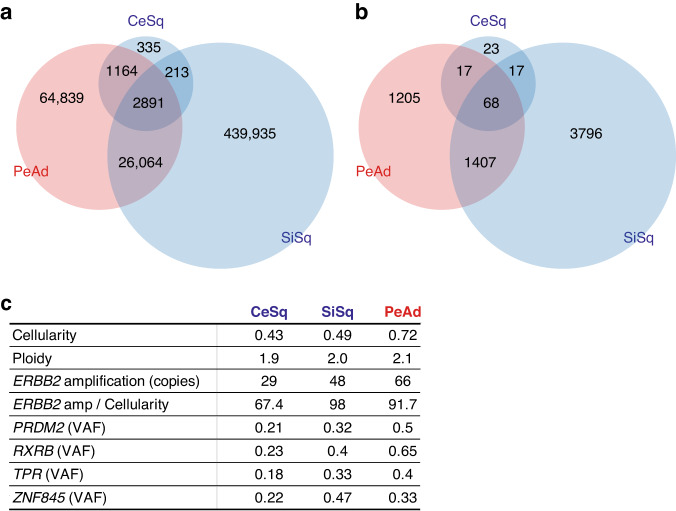
Whole genome sequencing analysis in the three lesions. **a** A Venn diagram indicating the number of mutations in CeSq, SiSq, and PeAd and their containment relationships. **b** A Venn diagram indicating the number of copy number variations (≥4 copies) in CeSq, SiSq, and PeAd and their containment relationships. **c** Summary of the functional analysis of whole genome sequencing. Functionally deleterious mutations of *PRDM2*, *RXRB*, *TPR*, and *ZNF845* are shared among CeSq, SiSq, and PeAd. Their variant allele frequencies (VAFs) in each specimen are shown in the table.

**Fig. 4:**
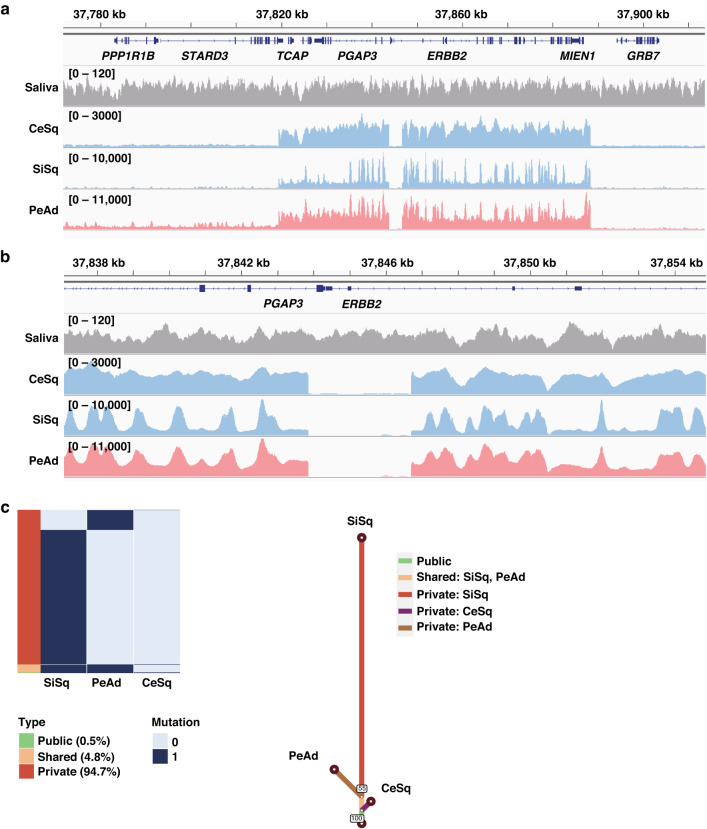
Amplification of *ERBB2* gene and integration of HPV-16 genome in the three lesions. Snapshots of the Integrative Genomics Viewer depicting *ERBB2*-amplification (**a**) and integration site of the HPV-16 genome (**b**). **c** The trajectory analysis based on mutation data. Mutations detected in at least one specimen were classified into “Public” (mutation detected in three specimens), “Shared” (mutation detected in two specimens), and “Private” (mutation detected in only one specimen) (left). The trajectory tree based on whole genome sequencing data (right).

Notably, not only the peritoneal adenocarcinoma but also the ileal lesion also showed the high expression of HER2 (Fig. 2b). Along with the fact that p16 was expressed in the peritoneal adenocarcinoma lesion in addition to the cervical and small intestinal squamous cell carcinoma lesions (Fig. 1c), these results suggest that these squamous cell carcinoma and adenocarcinoma lesions may have a common origin. Based on this hypothesis, we performed WGS of the cervical squamous cell carcinoma (CeSq) lesion, the small intestinal squamous cell carcinoma (SiSq) lesion, and the peritoneal adenocarcinoma (PeAd) lesion in order to analyze the differentiation trajectory of these lesions. The total number of mutations was 4603 in the CeSq lesion, 469,103 in the SiSq lesion, and 94,958 in the PeAd lesion (Fig. 3a). Most of the mutations detected in the CeSq lesion were also found in the SiSq and PeAd lesions. Although SiSq and PeAd shared 28,955 mutations, each had acquired unique mutations independently. The results of the copy number variation analysis showed similar trends (Fig. 3b). It is noteworthy that, in addition to the amplification of *ERBB2*, QIAGEN Clinical Insight® identified “likely pathogenic” mutations in *PRDM2* (PR domain zinc finger protein 2; c.3259C>T; [17, 18]), *RXRB* (retinoid X receptor beta; c.1174C>T), *TPR* (translocated promoter region, nuclear basket protein; c.6955C>T), and *ZNF845* (zinc finger protein 845; c.1831A>T) from 2891 mutations shared in the three lesions (Figs. 3c and 4a), which corroborates the hypothesis that these lesions originated from the same tumor cells. To investigate the possible involvement of human papillomavirus (HPV), we extracted sequence reads containing the HPV genome from the tumor genome and searched for the type of HPV and integration site in the human genome (Supplementary Table S1). A partial sequence of the HPV-16 genome was identified in the three lesions, and was found to be integrated into the same position on chromosome 17q12 (Fig. 4b, Supplementary Table S1).

An additional analysis of tumor evolution using mutation data revealed that CeSq, SiSq, and PeAd diverged from a trunk of original tumor cells, with the branch of PeAd being relatively short in comparison to that of SiSq (Fig. 4c). These data imply that the metastatic lesions developed from a trunk lesion prior to the divergence of CeSq, and that transdifferentiation from squamous cell carcinoma to adenocarcinoma was caused by an undetermined mechanism, such as acquired mutations and/or epigenetic alterations, most likely after the divergence of the seeds of PeAd during the progression of cancer cells.

To investigate the potential involvement of somatic mutations in transdifferentiation, we searched for coding and splice site mutations in PeAd but not in CeSq or SiSq. Of the 64,839 specific mutations in PeAd, 11 were localized in coding regions or at a splice site. The 11 comprised of four synonymous, six missense, and one splice site mutations. The six nonsynonymous mutations included DCST1 (NM_001143687), c.1447G>A (p.Gly483Arg); DDI1 (NM_001001711), c.1054G>A (p.Gly352Ser); SIAE (NM_170601), c.665C>T (p.Pro222Leu); KIF18B (NM_001264573), c.1039C>T (p.Arg347Trp); SEZ6 (NM_001098635), c.619G>A (p.Gly207Arg); P2RX3 (NM_002559), c.203G>A (p.Gly68Glu). Regarding the splice site mutation (REEP3, NM_001001330, c.106-1G>A), this change is likely to cause exon skipping or usage of an altered splice site because the mutation creates a new splice-acceptor motif with G at the 5’-end of the subsequent exon. However, since none of these seven genes have been reported to associate with carcinogenesis or differentiation, it remains unclear whether these mutations are involved in the transdifferentiation of the peritoneal metastasis. Other possibilities include epigenetic changes in the tumor during cancer progression.

## Discussion

We encountered a case in which squamous cell carcinoma and adenocarcinoma were concurrently diagnosed. A WGS analysis revealed that both tumors exhibited *ERBB2* amplification and HPV-16 genome sequences were identified at identical genomic loci. A trajectory analysis utilizing WGS data indicated that these cancers had a shared origin and that the squamous cell carcinoma transdifferentiated into adenocarcinoma. The recurrence of the disease was successfully managed with anti-HER2 therapy.

In general, when multiple tumor lesions are detected on imaging and the histological findings (e.g., hematoxylin-eosin staining or immunohistochemistry) of the lesions do not match, it is often assumed that the multiple lesions had independently developed with each other, and the diagnosis of multiple primary cancers is made. It is well known that genetic and environmental factors may play an important role in the development of multiple tumors. For example, pathogenic germline variants in mismatch repair genes predispose individuals with a wide range of neoplasms, including colorectal, endometrial, and gastric cancers [19], and in addition to being associated with the development of nasopharyngeal and lung cancer, tobacco smoking is associated with a high risk of esophageal cancer. It is also possible that multiple primary cancer tissues may be derived from cancer cells of the same origin. Several mechanisms have been proposed to explain the development of multiple histological types of cancer cells from the same origin. One possibility is the existence of “immature” cancer cells, from which each tissue type arises. Several in vitro and in vivo studies have confirmed the presence of a small population of cancer cells with cancer-initiating capacity, leading to the concept of “cancer stem cells” that are similar to tissue stem cells [20, 21]. Another possibility is that some cancer cells with one histological type change to those with different histological type(s). Several reports have shown that re-biopsy of tumor tissues revealed histological types that differed from those identified in the original biopsy [22–24]. Additionally, it is known that epithelial tumor cells can sometimes display mesenchymal characteristics (epithelial-mesenchymal transition; [25]). Importantly, these two mechanisms are not necessarily mutually exclusive.

A squamous cell carcinoma marker, p40, was highly expressed in the small intestinal lesion but not in the peritoneal lesion, while GATA3 and CAM5.2 were only expressed in the peritoneal lesion (Fig. 1c). GATA3 is a sensitive and relatively specific biomarker of urothelial and breast carcinomas, while most Müllerian carcinomas are GATA3-negative [26]. Based on this finding, we initially considered that the peritoneal lesion was not metastasis of cervical squamous cell carcinoma. However, we found that p16 and HER2 were highly expressed not only in the disseminated peritoneal adenocarcinoma lesion but also in the small intestinal squamous cell carcinoma lesion (Figs. 1c and 2b). According to the WGS analysis, the majority of the mutations detected in the uterine cervical carcinoma lesion were also found in both the small intestinal squamous cell carcinoma lesion and the peritoneal adenocarcinoma lesion. Furthermore, HPV-16 genome sequences were identified at the same genomic loci among three samples. These results indicate that the squamous cell carcinoma and adenocarcinoma in our case were derived from cancer cells of the same origin. We also observed that the integration was accompanied by a ~3 kb deletion including the 5’-untranslated region of the *ERBB2* gene and a part of the *PGAP3* gene (Fig. 4b). As the deleted region was completely lost in the three lesions, the integration of the viral genome and loss of the region may precede the amplification. Trajectory analysis based on mutation sites revealed that squamous cell carcinoma branched out in the direction of adenocarcinoma. Overall, our results suggest that a subpopulation of the cervical squamous cell carcinoma transdifferentiated into the adenocarcinoma in our patient and provide clinical evidence of tumor cell plasticity.

As WGS becomes more widely available, the number of cases in which multiple lesions, which were previously thought to be independent tumors, are shown to have the same origin may increase with comprehensive analyses through WGS, leading to a better understanding of tumor evolution and targeted therapies. In summary, our findings demonstrate the plasticity of tumor cells and reinforce the potential roles of WGS in the implementation of precision medicine for cancer.

## Supplementary information

Supplementary materials

Table S1

## Data Availability

All data generated or analyzed during this study are included in this published article and its supplementary information files.
