# Supplementary material for: Transdifferentiation of cervical squamous cell carcinoma with ERBB2 amplification to adenocarcinoma: whole genome sequence analysis and successful control by anti-HER2 therapy
Source: BJC Rep. 2023 Sep 4;1:12. doi: 10.1038/s44276-023-00015-9 (PMC11523955; doi:10.1038/s44276-023-00015-9)
Supplement: Supplementary file 1 — Supplementary materials [file 44276_2023_15_MOESM1_ESM.docx]

**Supplementary materials**

**Table Legends**

**Table S1.**

The number of HPV-16 genome reads detected in control saliva, CeSq, SiSq, and PeAd. Chromosomal position and read counts are listed. The major integration sites, highlighted with red or blue, were shared by the three specimens. Single_evidence: soft-clipped reads that are aligned to the HPV genome. Paired_evidence: read pairs with one read aligning to the human genome and the other to the HPV genome.
