# Supplementary material for: Transdifferentiation of cervical squamous cell carcinoma with ERBB2 amplification to adenocarcinoma: whole genome sequence analysis and successful control by anti-HER2 therapy
Source: BJC Rep. 2023 Sep 4;1:12. doi: 10.1038/s44276-023-00015-9 (PMC11523955; doi:10.1038/s44276-023-00015-9)
Supplement: Supplementary file 2 — Table S1 [file 44276_2023_15_MOESM2_ESM.pdf]

**Table S1**

| Saliva | chr | pos | single_evidence | paired_evidence |
|---|---|---|---|---|
| | 9 | 32063400 | 1 | 1 |
| CeSq | chr | pos | single_evidence | paired_evidence |
| | 17 | **39690467** | 495 | 1130 |
| | 17 | **39687597** | 430 | 1055 |
| | 17 | 39690523 | 28 | 1158 |
| | 17 | 39668240 | 7 | 24 |
| | 17 | 39690504 | 4 | 1152 |
| | 17 | 39666467 | 3 | 6 |
| | 17 | 39690470 | 3 | 1132 |
| | 1 | 191932688 | 2 | 2 |
| | 17 | 39668243 | 2 | 24 |
| | 17 | 39703001 | 2 | 2 |
| SiSq | chr | pos | single_evidence | paired_evidence |
| | 17 | **39687597** | 244 | 393 |
| | 17 | **39690467** | 207 | 299 |
| | 17 | 39679055 | 11 | 13 |
| | 17 | 39687355 | 3 | 205 |
| | 10 | 102504576 | 2 | 2 |
| | 10 | 129529885 | 2 | 2 |
| | 10 | 12960189 | 2 | 2 |
| | 10 | 85547392 | 2 | 2 |
| | 11 | 12724788 | 2 | 2 |
| | 1 | 114081003 | 2 | 2 |
| PeAd | chr | pos | single_evidence | paired_evidence |
| | 17 | **39690467** | 815 | 1446 |
| | 17 | **39687597** | 716 | 1386 |
| | 17 | 39690523 | 42 | 1457 |
| | 17 | 39669244 | 7 | 20 |
| | 17 | 39674335 | 6 | 15 |
| | 17 | 39690472 | 4 | 1449 |
| | 1 | 69026042 | 3 | 3 |
| | 17 | 39687544 | 3 | 1414 |
| | 17 | 39690470 | 3 | 1447 |
| | X | 31817587 | 3 | 2 |
